# Robotic-Assisted Joint Line Preserving Unicompartmental Knee Arthroplasty Positioning Among Octogenarians

**DOI:** 10.3390/jpm15080362

**Published:** 2025-08-08

**Authors:** Filippo Leggieri, Fernando Nahuel Martín Cocilova, Alessandro Civinini, Davide Stimolo, Roberto Civinini, Matteo Innocenti

**Affiliations:** Department of Clinical Orthopaedic, University of Florence, A.O.U. Careggi CTO, 50139 Florence, Italy

**Keywords:** medial unicompartmental knee arthroplasty, octogenarians, surgical technique, resurfacing, kinematic alignment, aseptic loosening

## Abstract

**Introduction**: Octogenarians undergoing unicompartmental knee arthroplasty (UKA) face increased risks of complications due to reduced bone support following osteoporosis. The aim of this study was to describe our preferred technique to balance robotic-assisted UKA in this specific patient population and to present its results. **Methods**: This retrospective analysis of prospectively collected data examined 121 consecutive octogenarian patients (median age 84 years, IQR 82–86) who underwent robotic-assisted medial UKA between September 2018 and December 2022 with ≥24 months follow-up. Patients aged ≤80 years, with ≤2 years of follow-up, or without informed consent were excluded. Data collection included radiographic measurements (HKA, LDFA, MPTA, joint line height), patient-reported outcome measures (Oxford Knee Score, Knee Society Score), and complications. Statistical analysis employed descriptive statistics, paired *t*-tests, Cohen’s d for effect sizes, and the McNemar test for categorical variables. **Results**: The hip–knee–ankle angle improved significantly from 174.43° to 178.04° (mean difference 3.61°, 95% CI 3.13–4.09, *p* < 0.001). Patient-reported outcomes demonstrated substantial improvements: the Knee Society Score increased by 83.09 points (95% CI 79.76–86.42, *p* < 0.001, Cohen’s d = 4.53), and the Oxford Knee Score increased by 17.09 points (95% CI 15.42–18.76, *p* < 0.001), with both exceeding minimal clinically important differences. Only 7.4% (9/121) of cases exhibited joint line lowering of >2 mm, with 1.7% (2/121) having both post-operative HKA <175° and joint line lowering of >2 mm. The implant survival rate was 100% with minimal complications, including two conservatively managed tibial plateau fractures and two cases of wound dehiscence with no further surgery needed. **Conclusions**: Robotic-assisted medial UKA can consistently preserve joint line height while achieving excellent alignment correction and clinical outcomes in octogenarians, potentially addressing failure risks in this specific population.

## 1. Introduction

Patients over 80 years old undergoing total knee arthroplasty (TKA) face higher risks of surgical and medical complications compared to younger patients, primarily due to their greater number of coexisting health conditions and reduced bone density [1]. For elderly patients with osteoarthritis limited to the medial compartment of the knee, medial unicompartmental knee arthroplasty (UKA) may offer significant advantages [2,3], including fewer complications, shorter surgery duration, and reduced hospitalization periods [4,5].

However, concerns exist regarding UKA’s long-term performance in octogenarians. Their characteristic osteoporotic bone, combined with UKA’s smaller footprint (relative to TKA), limited cortical support, and absence of a tibial keel potentially increase risks of tibial fractures or component loosening [6,7,8]. These risks can be substantially reduced through meticulous surgical techniques that ensure proper alignment and implant positioning [9], while avoiding joint line depression of 2mm or more in cases where the hip–knee–ankle (HKA) angle measures 175° or less [10].

A lowered joint line typically results from combining a femoral implant in superstructure with excessive tibial bone removal to accommodate the UKA. This places the bearing at a lower position where bone quality is poorer [11,12]. The combination of extensive tibial cutting and increased mechanical load caused by a reduced post-operative HKA can theoretically result in a higher rate of tibial implant failure [13].

The primary aim of this study was to describe our preferred technique to balance robotic-assisted UKA and consistently avoid joint line depression of 2 mm or more in cases where the hip–knee–ankle (HKA) angle measures 175° or less. The secondary aim was to present the results of our series of robotic-assisted UKAs among octogenarians.

## 2. Materials and Methods

### 2.1. Study Design and Cohorts

This study presents a retrospective analysis of prospectively collected data from robotic-assisted mUKA performed at a single institution between September 2018 and December 2022.

The inclusion criteria included patients aged ≥ 80 years old and with follow-up ≥ 24 months. Participants who did not provide consent to data collection were excluded. After screening 408 knees in our database against the inclusion and exclusion criteria, a total of 121 were deemed eligible for the analysis in the study with a median age of 84 years (IQR, 82 to 86).

### 2.2. Surgical Technique

Pre-operative assessment included standard weightbearing full-length lower-extremity radiographs along with CT scans following the MAKOPlasty protocol. These images were processed through STRYKER’s software platform (Stryker, Mahwah, NJ, USA) to create personalized virtual knee models for each patient’s surgical planning. In all cases, the same implant design was utilized—a fixed-bearing, metal-backed cemented unicompartmental prosthesis (Restoris MCK partial knee, Stryker, Mahwah, NJ, USA).

All procedures were performed with patients positioned supine utilizing MAKO^®^ robotic-assisted technology (Stryker^®^, Mahwah, NJ, USA). The standardized surgical sequence included tourniquet application, prophylactic antibiotic administration, and a consistent mini medial parapatellar approach. The tibial array was positioned at least 5 cm distal to the inferior border of the tibial tuberosity to prevent impingement with the proximal femoral array during knee extension. The femoral array was positioned intra-articular within the arthrotomy extremities, 2 cm proximal to the distal lateral femoral condyle, and inserted at a 45° inclination relative to the anterior cortex. This array configuration provided optimal visualization of the medial compartment through consistent retraction of the quadriceps muscle laterally. Femoral pins were not required, as registration was achieved via direct referencing from the femoral array.

The tibial rotation was set based on femoral anatomy as previously described and orientated at 0° relative to the femur-based rotation landmark [14]. The appropriate tibial component size was then determined, with fine-tuning of tibial rotation to prevent component overhang or underhang.

The tibial component was designed as a true pre-arthritic resurfacing procedure, emphasizing meticulous preservation of the joint’s native anatomy. A predetermined tibial component proudness of 4 mm was established to maintain physiological tibial anatomy while preventing depression of the joint line. When tibial component proudness below 4 mm was required to achieve optimal balancing, the overall coronal alignment was adjusted to ensure the final alignment did not fall below 175°. As the proudness was reduced, the residual varus was correspondingly decreased, while never exceeding 180° to avoid overloading the lateral compartment and risking osteoarthritic progression. This alignment adjustment was implemented to prevent overloading of the medial compartment when the tibial component was positioned deeper into the softer cancellous bone.

Additional alignment parameters were configured to restore the native posterior slope, with a maximum allowable slope of 7°. Exceptions were made exclusively for patients with isolated medial compartment osteoarthritis who presented with primary anteroposterior instability secondary to ACL insufficiency caused by progressive arthritic changes within the medial compartment. In these specific cases, where medial UKA remained the appropriate treatment option, a reduced posterior slope approaching 0° was deliberately implemented to achieve joint stability, even when the patient’s native anatomy would have warranted a greater slope angle. This modification allowed for successful medial UKA while avoiding the increased surgical complexity and perioperative risks associated with TKA in patients whose comorbidities made them suboptimal candidates for the more extensive procedure.

Balancing and pose acquisition were routinely assessed through manual evaluation, with rotational stability achieved by securing the femoral array with one hand while applying controlled valgus stress to the tibia with the contralateral hand. Component positioning was planned to provide greater looseness in extension within 2 mm and increased tightness in mid-flexion, without operating within restrictive boundaries since the patient’s native anatomy was preserved without alterations. Balancing in deep flexion was not considered in the surgical planning, as no evident advantage has been demonstrated for maintaining a closer-to-physiologic laxity compared to tighter or looser balancing [15].

Patellar tracking was assessed at the beginning of the procedure by establishing bony contact at the centre of the patella using electrocautery and acquiring reference points with the probe positioned at the patellar centre throughout the complete range of motion. An identical maneuver was repeated at the conclusion of the procedure to evaluate optimal patellar centring and trochlear engagement and to determine whether the anterior offset had been adequately restored.

### 2.3. Population and Follow-Up

Data such as age, body mass index (BMI), hip–knee–ankle (HKA) angle, mechanical lateral distal femur angle (mLDFA), and medial proximal tibia angle (MPTA) were recorded pre-operatively [16]. Post-operatively, the MPTA and LDFA were calculated relative to the respective mechanical axis of the tibia and the femur, respectively. The Oxford Knee Score (OKS) and the Knee Society Score (KSS) were assessed pre-operatively, as well as at 2-year follow-up [17,18]. The joint line height was calculated on AP knee X-rays according to Herry’s protocol [19] as the difference between the line perpendicular to the lateral cortex of the femur touching the native joint line of the distal lateral condyle and its parallel touching the femoral component on the distal medial condyle. Any intraoperative and post-operative complication was registered. Implant failure was defined according to the following criteria: any requirement for subsequent surgical intervention following the initial procedure, including implant removal and conversion to total knee arthroplasty; radiographic evidence of implant loosening; progression of osteoarthritis; or the necessity to perform either lateral UKA or patellofemoral arthroplasty on the same knee.

### 2.4. Data Analysis

Statistical analyses were performed using SPSS software version 29 (IBM Corporation, Armonk, NY, USA). Descriptive statistics including means, standard deviations, medians, and interquartile ranges were calculated for all continuous variables. Pre- and post-operative comparisons were conducted using paired *t*-tests, with Cohen’s d calculated to determine effect sizes. The McNemar test was employed to assess changes in HKA categories (≥175° vs. <175°) from pre-operative to post-operative status. Statistical significance was set at *p* < 0.05, and minimal clinically important differences (MCIDs) were evaluated for all patient-reported outcome measures.

## 3. Results

**Figure 1:**
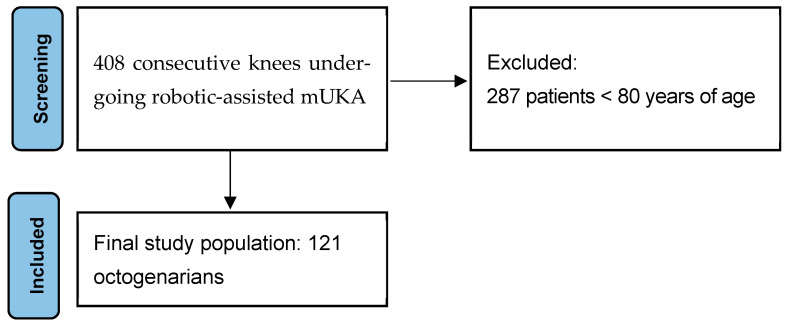
PRISMA flowchart of the population included.

The final study population consisted of 121 octogenarian patients who underwent the procedure (Figure 1). Baselines are provided in Table 1.

The hip–knee–ankle (HKA) angle demonstrated a statistically significant improvement from pre-operative to post-operative values, with a large effect size that exceeded minimal clinical importance (Table 2). No significant changes were observed in either the LDFA or MPTA while PROMs showed marked improvements post-operatively (Table 2).

From the HKA angle analysis assessment, 47.1% (57/121) of patients maintained a post-operative HKA angle ≥ 175°, while 46.3% (56/121) improved from <175° to ≥175°. Only 5.8% (7/121) of patients remained in the <175° category, and no patients (0.0%) experienced a decline from ≥175° to <175°. All 57 patients who presented with pre-operative HKA angles ≥ 175° maintained this alignment post-operatively. The McNemar test revealed a highly significant change in HKA angle categories following the intervention (χ^2^ = 54.018, df = 1, *p* < 0.001). The analysis of join line height measurements demonstrated favourable radiographic outcomes following the intervention. Only 7.4% (9/121) of cases exhibited a joint line lowering greater than 2 mm, but these cases were only marginally above the threshold, with joint line lowering values ranging from 2.1 to 2.2 mm. In these cases, tibial proudness values ranged from 3.7 to 4.0 mm. Only 1.7% of patients had both post-operative HKA < 175° and joint line lowering >2 mm (Table 3).

The survival rate at the final follow-up was 100%. There were two cases of wound dehiscence/leakage treated non-surgically and two cases of intracortical lateral tibial plateau fractures following a fall, both of which were managed conservatively without the need for surgical intervention. No additional complications were reported.

## 4. Discussion

This study demonstrates that our robotic-assisted medial UKA technique consistently preserves joint line height, with the surgeon having the option to decide whether to maintain HKA angles ≤ 175°. The results show that only 7.4% of cases exhibited a joint line greater than 2 mm, with only 1.7% having both post-operative HKA < 175° and joint line >2 mm. These findings support the efficacy of our preferred technique, which balances robotic-assisted UKA and consistently avoids joint line depression of 2 mm or more in cases where the HKA angle measures 175° or less. The prior literature has established that joint line depression of 2 mm or more in cases with lower alignment angles (HKA ≤ 175°) may significantly increase the risk of tibial component failure [10].

This relationship is due to the combined effect of increased mechanical load due to residual varus alignment and reduced bone quality at a depressed joint line position where the bone stock is softer [11,12].

Our technique prioritizes personalized planning for each patient to reconstruct native pre-arthritic joint surface anatomy [14]. The robotic-assisted approach facilitates precise implementation of this approach, resulting in consistent outcomes across our cohort. The 100% survival rate at final follow-up, along with minimal complications, reinforces the reliability of this method even in this high-risk population [20]. The implementation of the robotic platform maximizes the precision in the positioning of the component, thus favouring implant survival [21,22,23,24]. Robotic-assisted UKA was associated with reduced revisions for implant loosening, progression of disease, residual pain, and fracture, but increased revisions for infection compared to non-robotic UKA [25,26,27,28,29,30].

Manual UKA is a tibia-first procedure where deformity correction is primarily accomplished through the tibial cut, with femoral resurfacing offering limited options for fine-tuning component positioning and proudness. This constraint provides surgeons with minimal flexibility to make adjustments within the aforementioned parameters, potentially contributing to higher failure rates. This consideration is particularly significant given the established correlation between low surgical volume and increased failure rates in manual UKA procedures [31,32].

As expected, the significant improvements in patient-reported outcome measures (PROMs), including KSS (mean difference: 83.09, *p* < 0.001) and OKS (mean difference: 17.09, *p* < 0.001), both exceeding their respective MCIDs, indicate that our technique not only achieves favourable radiographic outcomes but also translates to meaningful clinical benefits for patients. In our case series, the two cases of intracortical lateral tibial plateau fractures were related to falls rather than implant-related issues and were successfully managed conservatively.

In this study, there was a dual rationale for choosing UKA in octogenarian patients. First, elderly patients have increased perioperative risk and greater rehabilitation difficulties. UKA reduces surgical burden and reduces post-operative rehabilitation burden compared to TKA, resulting in faster and less demanding surgical intervention, recovery and rehabilitation, which are particularly advantageous in this frail population where surgical disability must be minimized [33,34,35]. The other important consideration when performing UKA in octogenarian patients is the critical need for a definitive “one-shot” surgical solution that minimizes revision risk. Elderly patients cannot afford surgical failure requiring revision, as a failed UKA necessitating conversion to TKA would be significantly more disadvantageous than primary TKA—the revision procedure would be more invasive and occur when the patient was even older and potentially more frail. However, this concern must be balanced against established registry evidence demonstrating that UKA achieves equivalent very-long-term survival rates to TKA, particularly when performed by high-volume surgeons with robotic assistance for precise component positioning [31,32,36]. The rationale for choosing the less invasive UKA in elderly patients rests on the principle that if medium-to-long-term outcomes are equivalent between procedures, octogenarian patients may complete their natural lifespan with a well-functioning UKA given their limited life expectancy [37]. Our technical approach, emphasizing joint line preservation and optimal component positioning while avoiding contact with soft cancellous bone, is specifically designed to maximize UKA survivorship and address the common failure modes that might otherwise deter surgeons from offering this less invasive option to this vulnerable population.

This study specifically addresses optimized UKA techniques tailored for the octogenarian population, including the management of complex clinical scenarios that would traditionally contraindicate UKA procedures. In those cases where patients present with secondary ACL insufficiency—resulting from combined mechanisms of progressive cartilage thinning in the isolated medial compartment creating increased medial intra-articular gap and ACL structural compromise due to arthritis progression—the anteroposterior instability can be managed through personalized surgical modifications [38,39,40]. Our technique employs a deliberate reduction in tibial slope, even to near 0°, to achieve joint stability and compensate for ligamentous insufficiency, thereby resolving the mechanical instability that would otherwise advise against or preclude successful UKA. This technical adaptation allows surgeons to offer knee replacement to octogenarian patients with advanced symptomatic arthritis who would otherwise be denied surgical intervention due to objectively assessed instability and higher risk associated with the more extensive TKA procedure. By maintaining the less invasive nature of UKA while addressing specific biomechanical challenges through personalized surgical planning, this approach demonstrates that UKA can remain feasible and effective even in challenging clinical scenarios, ultimately improving quality of life in elderly patients with debilitating knee symptoms who have limited alternative treatment options.

Previous reports examining UKA outcomes in octogenarian patients demonstrate generally favourable results. Multiple large-scale studies have shown comparable outcomes between elderly and younger patients, including similar rates of complications, morbidity, readmission, reoperation, prosthetic joint infection, periprosthetic fractures, and aseptic loosening at 2-year follow-up [2,37,41,42,43,44]. In a recent metanalysis of RCTs comparing UKA and TKA for unicompartmental knee osteoarthritis, UKA demonstrated significantly fewer post-operative complications and shorter hospital stays compared to TKA, with equivalent survivorship, as evidenced by no significant differences in revision or failure rates between the procedures [45]. However, when applying minimal clinically important difference criteria, there were no clinically meaningful differences between the procedures in terms of knee recovery, function, pain relief, or patient satisfaction, suggesting that the primary advantages of UKA are improved safety and reduced hospitalization while achieving comparable long-term survivorship to TKA [45].

Thus, this represents the first study to specifically address and validate surgical techniques that can be systematically adapted to optimize outcomes in octogenarian patients using robotic-assisted technology. While the previous literature has demonstrated favourable outcomes among UKA patients, no prior investigation has comprehensively described the technical modifications and strategic surgical planning necessary to maximize implant survivorship in this high-risk population. Our study fills this critical gap by providing evidence- and experience-based technical guidelines for joint line preservation, alignment optimization, and management of complex clinical scenarios, establishing a reproducible surgical framework specifically tailored for the unique challenges presented by octogenarian patients undergoing robotic-assisted UKA.

This study has several limitations. The retrospective design, although based on prospectively collected data, inherently introduces potential selection bias. The single-cohort design without a comparative control group precludes any conclusion regarding superiority or definitive risk reduction compared to alternative surgical approaches. While the results suggest potential benefits in this high-risk population, the absence of comparative data limits the ability to report improved outcomes over conventional manual techniques or alternative robotic protocols. The 24-month follow-up period, while sufficient for early complications, is inadequate for detecting aseptic loosening, which typically occurs beyond two years post-operatively. This limitation prevents assessment of long-term implant survivorship and the technique’s effectiveness against this primary failure mode in elderly patients.

Future prospective comparative studies are required to validate any findings of improved outcomes and establish the relative efficacy of this technique compared to other approaches. Longer-term studies would be valuable to confirm the durability of these results.

## 5. Conclusions

Our robotic-assisted medial UKA technique consistently preserves joint line height while improving alignment in octogenarian patients. These favourable radiographic outcomes, coupled with significant improvements in PROMs and minimal complications, suggest that this approach may address the higher risk of failure in this high-risk population. The importance of avoiding joint line depression, particularly in cases with lower HKA angles, should be a key consideration in surgical planning for elderly patients undergoing UKA.

## Figures and Tables

**Table 1:** Baselines characteristics of the included population.

	Time	Mean	SD	Median	Min	Q25	Q75	Max
HKA	Pre-op	174.43	3.34	174	166	172	177	180
LDFA		88.25	1.6	88	84	87	90	92
MPTA		86.18	2.08	86	80	85	88	90
KSS		108.23	15	107	68	99	117	163
OKS		25.42	6.55	25	0	21	30	42
HKA	Post-op	178.04	2.08	179	169	177	180	182
LDFA		88.25	1.6	88	84	87	90	92
MPTA		86.28	1.68	86	81	85	87	90
KSS		191.32	12.2	195	128	189	200	200
OKS		42.51	5.54	45	24	40	46	48
Join line height lowering		0.4612	0.5	−1.1	−0.35	1.3	2.2	0.4612
Proudness in femur		2.38	0.38	2.4	1.5	2.2	2.4	5.2
Proudness in tibia		3.93	0.15	3.9	3.7	3.8	4.1	4.2

HKA, hip–knee–ankle angle; LDFA, lateral distal femoral angle; MPTA, medial proximal tibial angle; KSS, Knee Society Score; OKS, Oxford Knee Score; SD, standard deviation; Q25, 25th percentile/first quartile; Q75, 75th percentile/third quartile; Min, minimum value; Max, maximum value.

**Table 2:** Statistical comparison results.

	Pre-Op Mean	Post-Op Mean	Mean Diff.	95%CI	*p*	Cohen’s d	MCID	Exceeds MCID
HKA	174.43	178.04	3.61	3.13 to 4.09	<0.001	1.01	3	Yes
LDFA	88.25	88.08	−0.17	−0.50 to 0.16	0.317	−0.04	2	No
MPTA	86.18	86.28	0.1	−0.28 to 0.49	0.598	0.18	2	No
KSS	108.23	191.32	83.09	79.76 to 86.42	<0.001	4.53	20	Yes
OKS	25.42	42.51	17.09	15.42 to 18.76	<0.001	1.9	5	Yes

HKA, hip–knee–ankle angle; LDFA, lateral distal femoral angle; MPTA, medial proximal tibial angle; KSS, Knee Society Score; OKS, Oxford Knee Score; Pre-op Mean, pre-operative mean; Post-op Mean, post-operative mean; Mean Diff., mean difference; 95%CI, 95% confidence interval; *p*, *p*-value; Cohen’s d, effect size measure; MCID, minimal clinically important difference; Exceeds MCID, whether the mean difference exceeds the minimal clinically important difference.

**Table 3:** Distribution of the outliers.

	JLL ≤ 2 mm	JLL > 2 mm	Total
HKA ≥ 175°	51 (42.1%)	7 (5.8%)	58 (47.9%)
HKA < 175°	61 (50.4%)	2 (1.7%)	63 (52.1%)
Total	112 (92.5%)	9 (7.5%)	121 (100%)

HKA, hip–knee–ankle angle; JLL, joint line lowering.

## Data Availability

The dataset used and analyzed during the current study is available from the corresponding author on reasonable request.
